# Comparison of quality metrics in an education-centered medical home with local and national benchmarks

**DOI:** 10.1080/10872981.2022.2073806

**Published:** 2022-05-11

**Authors:** Ana Sofia Mesa, Marianne Tschoe

**Affiliations:** a Department of Family Medicine, Madigan Army Medical Center, Tacoma, Washington, USA; b Department of Internal Medicine, Northwestern University Feinberg School of Medicine, Chicago, Illinois, USA

**Keywords:** Free clinic, medical home, preventative care, longitudinal clerkship

## Abstract

The Education-Centered Medical Home (ECMH) is a longitudinal clerkship where students provide care to patients at one clinic site for the entirety of medical school. Studies have demonstrated that ECMHs have higher completion rates of preventative measures than traditional student-run free clinics (SRFCs). However, data comparing ECMHs with licensed primary care provider clinics are limited. We performed a prospective chart review that examined vaccination and cancer screening rates of patients in an ECMH and those seen by primary care physicians (PCPs) at the same free clinic site. We then compared these groups with participants in the 2018 National Health Information Study (NHIS). A total of 62 ECMH patients, 3,515 PCP patients, and 25,045 NHIS participants were included in the study. Within the ECMH, 72.7% and 80.0% of patients were screened for breast and cervical cancer, respectively. These rates did not differ significantly from those of PCP patients or NHIS participants. While the percentage of ECMH patients screened for colon cancer was similar to that of PCP patients (78.9% vs. 65.8%, p = 0.09), it was proportionally greater than NHIS screening rates (78.9% vs. 63.3%, p = 0.043). In addition, the rate of influenza and pneumococcal pneumonia vaccination among ECMH patients (41.4% and 58.3%, respectively) did not differ significantly from the PCP and NHIS groups. Our study found that the ECMH model allows students to deliver preventative care comparable to licensed practitioners and national benchmarks. It reaffirms the ECMH as an effective method for students to provide high quality care to underserved patients.

## Introduction

Student-run free clinics (SRFCs) provide essential services that would otherwise be inaccessible to uninsured or underinsured patients [1]. SRFCs have positively impacted such diverse measures as prevention, clinical outcomes, and resource utilization [2–9]. Yet, patient and provider discontinuity remains a barrier to quality improvement [7]. Continuity of care requires more organization and commitment than the traditional arrangement of SRFCs, where students and preceptors attend clinic on an ad hoc, rather than routine, basis.^1^

The Education-Centered Medical Home (ECMH) is a longitudinal clerkship that emphasizes continuity among students, preceptors, and patients in a primary care setting [10]. At each ECMH clinic site, a cohort of students provides care to the same patient panel for the duration of their medical school careers. Their supervising preceptors come from a variety of disciplines (family medicine, internal medicine, pediatrics) and practice settings, including Federally Qualified Health Centers and free clinics. After initiation of the ECMH model at some primary care practices, quality improvement data demonstrated performance gains in diabetic foot exams, chlamydia screening rates, medical attention to diabetic nephropathy, and use of inhaled steroids for moderate-severe persistent asthma [10]. Subsequent data at one site showed significant improvements in cancer screening and influenza vaccination after implementation of the ECMH model [11]. Additionally, when an SRFC was converted into an ECMH, it had higher completion rates across multiple preventive measures when compared with traditional SRFCs [12].

Yet, because of the differences in how they operate, benchmarking an ECMH with standard SRFCs may not accurately assess its overall performance. To better understand the impact of the ECMH model on quality improvement, we compared it with the traditional primary care delivered by licensed physicians in the same free clinic setting. These two models differ in provider level of training but share many other similarities, including using limited resources to serve uninsured patients as well as relying on continuity of care to help patients achieve their health goals. Because they share similar patient populations, clinic setting, and commitment to continuity, we hypothesized that overall performance on preventive health measures should also be similar. We compared vaccination and cancer screening rates between the two groups to test this hypothesis. We also evaluated both against national health survey data to assess how they compared with average care. A lack of significant performance differences between the ECMH SRFC and national benchmarks would further support the importance of continuity in improving the quality of care at university-affiliated SRFCs.

## Methods

### Setting

*CommunityHealth Chicago*: CommunityHealth Chicago (CHC) is the largest volunteer-based free medical facility in the nation. It provides primary and specialty healthcare to patients who cannot afford and do not qualify for insurance, including state and federal programs. CHC relies on individual, foundational, institutional, and corporate donations to finance its operations as a free clinic. It does not receive any funding from government programs or grants. CHC houses multiple primary care clinics and SRFCs at its location, including the ECMH SRFC in this study.

*Education-Centered Medical Home*: ECMH is a longitudinal clinical clerkship where students participate in patient care at one clinic site for the entirety of their medical school careers. Core ECMH principles include ‘continuity with a personal physician; team-based care; care coordination and integration; quality and safety; and enhanced access to care’ [13]. ECMH students attend clinic approximately twice per month. They work with the same physician preceptor each time. Schedule assignments emphasize continuity between individual students and patients so that each sees the other as ‘their patient’ or ‘their provider.’ Typically, pairs of students engage in patient encounters with physician preceptor oversight. Students are expected to actively participate in all aspects of care, including patient communication, documentation in the electronic medical record, evidence-based clinical decision-making, and follow-up. Preceptors must approve all management and clinical decisions [14].

*Primary Care Clinics*: Providers in CHC’s primary care clinics (PCP) are graduates of professional schools. They are either fully licensed to practice medicine, such as physicians and advanced practice providers, or licensed to practice under the supervision of a training program. All PCPs have a set group of patients assigned to them.

### Study design

This was a prospective chart review of quality measure completion rates for patients seen by the ECMH and 82 PCP clinics at CHC. Results from each group were compared with each other and with data from the 2018 National Health Information Survey (NHIS) [15]. Outcomes for quality measures were defined as follows:

- Colon cancer screening: Fecal occult blood immunoassay of a stool sample in the last year, flexible sigmoidoscopy in the previous five years, or colonoscopy in the previous ten years [16]
- Breast cancer screening: Mammogram in the last two years [17]
- Cervical cancer screening: Pap smear within the past three years or a documented Pap smear with co-testing for high-risk HPV within the past five years [18]
- Immunization against influenza: Influenza vaccination within the previous year [19]
- Immunization against pneumonia: Vaccination with 23-valent pneumococcal polysaccharide vaccine (PPSV23) or 13-valent pneumococcal conjugate vaccine (PCV13) according to guidelines from the Advisory Committee on Immunization Practices [20,21]

### Study population

Patients at CHC do not have access to any form of health insurance. Additionally, their income must not exceed 250% of the federal poverty line. Patients were included in this study if they met eligibility criteria for any of the following quality measures:

- Colon cancer screening: adults aged 50 to 75 [16]
- Breast cancer screening: women aged 50 to 74 [17]
- Cervical cancer screening: women aged 30 to 64 [18]
- Immunization against influenza: adults aged 19 and older [19]
- Immunization against pneumonia: adults aged 65 and older or adults with immunocompromising conditions [20,21]

### Data collection

Data for the ECMH and PCP groups were collected in April 2020 using the ‘Quality Report’ function on the Athena® Electronic Medical Record (EMR). The EMR classifies patients as having satisfied a metric based on records of completed test results and vaccinations. Data from each quality report was de-identified and stored in a secure folder on the Box™ file-sharing system licensed by Northwestern University.

Publicly available NHIS data collected in 2018 was downloaded from the Centers for Disease Control and Prevention National Center for Health Statistics [15]. Northwestern University’s Institutional Review Board determined that the study protocol did not meet criteria for human subjects research.

### Data analysis

Data analysis was performed using Microsoft Excel®. Two-sided p-values for significance were calculated using chi-squared analyses; p-values of less than 0.05 were considered significant.

## Results

### Patients

A total of 62 ECMH patients, 3,515 PCP patients, and 25,045 NHIS participants were included. Each group had variations between baseline characteristics (Table 1). On average, ECMH patients tended to be older, with the mean (±SD) age of 55 (±11.9) as compared to a mean age of 48.9 (±13.4) in PCP and 51.7 (± 18.3) in NHIS patients. A majority of the patients from CHC were female (2170 [60.7%]) and identified as Hispanic (2430 [67.9%]). However, only a minority of NHIS participants identified as Hispanic (2807 [11.2%]). Additionally, the ECMH patients and NHIS participants were more likely to report their race as Caucasian (26 [41.9%] and 20,414 [80.3%], respectively) as compared to PCP patients (967 [27.8%]). Finally, NHIS participants were much more likely to report their race as Black or African American (3101 [12.2%]) than CHC patients.

**Table 1.** Patient Characteristics

Characteristics	ECMH	PCP	NHIS
**Age –** yr (± SD)	55 (± 11.9)	48.9 (± 13.4)	51.7 (± 18.3)
**Sex –** no. (%)			
Male	24 (38.7)	1383 (39.3)	11,550 (45.4)
Female	38 (61.3)	2132 (60.7)	13,867 (54.6)
**Race –** no. (%)			
African American	0 (0.0)	157 (4.5)	3101 (12.2)
American Indian	0 (0.0)	4 (0.1)	360 (1.4)
Asian	0 (0.0)	82 (2.4)	943 (3.8)
Pacific Islander	0 (0.0)	5 (0.1)	-
Caucasian	26 (41.9)	967 (27.8)	20,414 (80.3)
Other	29 (46.8)	1414 (40.6)	79 (0.3)
Declined	7 (11.3)	850 (24.4)	85 (0.3)
**Ethnicity –** no. (%)			
Hispanic	41 (66.1)	2389 (68.0)	2807 (11.2)
Not Hispanic	21 (33.9)	1126 (32.0)	22,238 (88.8)
*Total*	*62*	*3515*	*25,045*

*ECMH Patients in the education-centered medical home, a longitudinal clerkship model where students participate in patient care at one clinical site for the entirety of their medical careers under the supervision of the same physician preceptorPCP Patients in traditional primary care models with a single provider who is either a physician, advanced practice practitioner, or licensed trainee within a supervised training program.NHIS Participants in the 2018 National Health Survey collected by the Centers for Disease Control and Prevention*

### Outcomes

Within the ECMH, 16 (72.7%) and 24 (80.0%) patients were screened for breast and cervical cancer, respectively (Table 2). These rates did not differ significantly from those of PCP patients or NHIS participants (p = 0.187). While the percentage of ECMH patients screened for colon cancer was similar to that of PCP patients (78.9% vs. 65.8%, p = 0.09), it was proportionally greater than NHIS screening rates (78.9% vs. 63.3%, p = 0.043). Finally, screening rates for influenza did not differ significantly between the ECMH and PCP groups (41.4% vs 36.1%, p = 0.408) or between ECMH and NHIS (41.4% vs 47.6%, p = 0.343). Similar findings were also demonstrated in the screening rates for pneumonia (58.3% vs 74.2% for PCP (p = 0.219) and 68.5% for NHIS (p = 0.447).

**Table 2.** Preventative measure completion rate

P values represent differences between the ECMH group and either the PCP or NHIS group, respectively.		
Measure	No. (%)	P-Value
Breast Cancer		
ECMH	16 (72.7)	-
PCP	538 (58.7)	0.187
NHIS	3422 (63.3)	0.361
Cervical Cancer		
ECMH	24 (80.0)	-
PCP	1276 (73.8)	0.440
NHIS	5790 (80.0)	0.974
Colon Cancer		
ECMH	30 (78.9)	-
PCP	1022 (65.8)	0.090
NHIS	7034 (63.1)	0.043
Influenza Vaccine		
ECMH	24 (41.4)	-
PCP	1193 (36.1)	0.408
NHIS	11924 (47.6)	0.343
Pneumonia Vaccine		
ECMH	7 (58.3)	-
PCP	333 (74.2)	0.219
NHIS	4842 (68.5)	0.447

*ECMH Patients in the education-centered medical home, a longitudinal clerkship model where students participate in patient care at one clinical site for the entirety of their medical careers under the supervision of the same physician preceptor PCP Patients in traditional primary care models with a single provider who is either a physician, advanced practice practitioner, or licensed trainee within a supervised training program. NHIS Participants in the 2018 National Health Survey collected by the Centers for Disease Control and Prevention*

## Discussion

Across all preventative measures evaluated, the screening and vaccination rates of patients receiving care through the ECMH were comparable to the rates of patients cared for by licensed practitioners. The ECMH and PCP clinics observed in this study are both housed within the same free clinic setting and draw from the same pool of patients. This comparison allowed PCPs to serve as a benchmark while controlling for confounding factors. By demonstrating care on par with the local standard, the ECMH model not only allowed students to learn from their experiences but also to have a meaningful and measurable impact on preventative care. In addition, patients who seek care at free clinics like CHC are uninsured, low-income, and predominantly from minority ethnic and racial backgrounds. These vulnerable health care populations stand to benefit the most from the continuity that the ECMH model can add to SRFCs. The ECMH’s coordination of students, preceptors and patients does require more organization and management than that of the traditional university-affiliated SRFC. Our study indicates that the additional effort could yield clear benefits to patient outcomes.

Additionally, screening rates of ECMH patients matched those of patients in the nationwide NHIS study, which includes insured populations. Several studies have previously shown that SRFC quality metrics can meet or exceed national benchmarks [2,5–9,11]. Our study supports the existing evidence that SRFCs can deliver care to underserved populations comparable to that received by average Americans. The standard of care can occur even in the face of significant barriers, such as low socioeconomic status and lack of insurance.

## Limitations

Our study has several limitations, the most significant of which is the large variation in sample size. There were a total of 62 eligible patients within the ECMH, and sample sizes were further reduced within some preventative care categories based on inclusion criteria. This is most notable in our pneumonia vaccination group, which only included seven patients due to the age requirement. Conversely, the PCP clinic patients and NHIS survey participants numbered in the hundreds and thousands, respectively. This severely limits the conclusions that can be drawn from our data, particularly as the effect size is unknown. Ideally, our findings would be a starting point for further studies on the effectiveness of the ECMH model. Additionally, the ‘Quality Report’ function on the Athena® Electronic Medical Record only allowed for the collection of current quality measure satisfaction and did not allow for the review of prior rates of vaccination and cancer screening completion. However, as completion rates for the quality measures included in this study required, at most, annual completion, a single snapshot in time should have been sufficient to capture satisfaction rates. Finally, there were significant differences in the racial and ethnic make-up of the different groups included in this study. For example, ECMH patients were more likely to identify as Caucasian than PCP patients. As race underlies significant health disparities within the USA, the larger percentage of non-White patients within PCP-led clinics may have created an additional barrier to care.

## Conclusions

Student-run free clinics serve an important role as primary care providers to our nation’s most at-risk populations. The ECMH model achieves effective care delivery in the SRFC setting by emphasizing continuity as part of its mission. Establishment of ECMH as a longitudinal clerkship facilitates this goal. Yet, many SRFCs are not currently integrated into their schools’ curricula. Future studies could examine the feasibility of incorporating continuity of students, preceptors, and patients into SRFCs for which this is not currently the standard. These investigations could help determine if integration into the curriculum is necessary to attain continuity as well as measure impacts on preventative care. Ultimately, patients accessing free clinics must surmount significant barriers to health and healthcare. This study provides evidence that continuity can aid SRFCs in delivering high-quality primary care to our most vulnerable patients.

## Data Availability

Deidentified data collected from Community Health Chicago are available from the corresponding author upon reasonable request. The 2018 National Health Information Study data are available in the public domain at https://www.cdc.gov/nchs/nhis/nhis_2018_data_release.html. Code availability: Microsoft Excel was used for all data analysis. No custom codes were utilized.
